# Risk score to stratify miscarriage risk levels in preconception women

**DOI:** 10.1038/s41598-021-91567-8

**Published:** 2021-06-08

**Authors:** Xin Hui Choo, Chee Wai Ku, Yin Bun Cheung, Keith M. Godfrey, Yap-Seng Chong, Lynette Pei-Chi Shek, Kok Hian Tan, Thiam Chye Tan, Sadhana Nadarajah, Fabian Kok Peng Yap, Marjorelee T. Colega, Mary Foong-Fong Chong, Shiao-Yng Chan, See Ling Loy, Jerry Kok Yen Chan

**Affiliations:** 1 Yong Loo Lin School of Medicine, National University of Singapore, National University Health System, Singapore, 119228 Singapore; 2 Duke-NUS Medical School, Singapore, 169857 Singapore; 3 Department of Obstetrics and Gynaecology, KK Women’s and Children’s Hospital, Singapore, 229899 Singapore; 4 Program in Health Services & Systems Research and Center for Quantitative Medicine, Duke-NUS Medical School, Singapore, 169857 Singapore; 5 Tampere Center for Child, Adolescent and Maternal Health Research, Tampere University, 33014 Tampere, Finland; 6 Medical Research Council Lifecourse Epidemiology Unit, University of Southampton, Southampton, SO16 6YD UK; 7 National Institute for Health Research Southampton Biomedical Research Centre, University of Southampton and University Hospital Southampton National Health Service Foundation Trust, Southampton, SO16 6YD UK; 8 Singapore Institute for Clinical Sciences, Agency for Science, Technology and Research (A*STAR), Singapore, 117609 Singapore; 9 Department of Paediatrics, Yong Loo Lin School of Medicine, National University of Singapore, National University Health System, Singapore, 119228 Singapore; 10 Khoo Teck Puat-National University Children’s Medical Institute, National University Hospital, National University Health System, Singapore, 119074 Singapore; 11 Department of Maternal Fetal Medicine, KK Women’s and Children’s Hospital, Singapore, 229899 Singapore; 12 Department of Reproductive Medicine, KK Women’s and Children’s Hospital, 100, Bukit Timah, Singapore, 229899 Singapore; 13 Department of Paediatrics, KK Women’s and Children’s Hospital, Singapore, 229899 Singapore; 14 Saw Swee Hock School of Public Health, National University of Singapore, National University Health System, Singapore, 117549 Singapore; 15 Department of Obstetrics and Gynaecology, Yong Loo Lin School of Medicine, National University of Singapore, National University Health System, Singapore, 119228 Singapore

**Keywords:** Epidemiology, Reproductive disorders, Risk factors

## Abstract

Spontaneous miscarriage is one of the most common complications of pregnancy. Even though some risk factors are well documented, there is a paucity of risk scoring tools during preconception. In the S-PRESTO cohort study, Asian women attempting to conceive, aged 18-45 years, were recruited. Multivariable logistic regression model coefficients were used to determine risk estimates for age, ethnicity, history of pregnancy loss, body mass index, smoking status, alcohol intake and dietary supplement intake; from these we derived a risk score ranging from 0 to 17. Miscarriage before 16 weeks of gestation, determined clinically or via ultrasound. Among 465 included women, 59 had miscarriages and 406 had pregnancy ≥ 16 weeks of gestation. Higher rates of miscarriage were observed at higher risk scores (5.3% at score ≤ 3, 17.0% at score 4–6, 40.0% at score 7–8 and 46.2% at score ≥ 9). Women with scores ≤ 3 were defined as low-risk level (< 10% miscarriage); scores 4–6 as intermediate-risk level (10% to < 40% miscarriage); scores ≥ 7 as high-risk level (≥ 40% miscarriage). The risk score yielded an area under the receiver-operating-characteristic curve of 0.74 (95% confidence interval 0.67, 0.81; p < 0.001). This novel scoring tool allows women to self-evaluate their miscarriage risk level, which facilitates lifestyle changes to optimize modifiable risk factors in the preconception period and reduces risk of spontaneous miscarriage.

## Introduction

Spontaneous miscarriage is one of the most common complications of pregnancy, occurring in 12–26% of recognized pregnancies^1^. Spontaneous miscarriage is multifactorial in aetiology, with biological, environmental, obstetric and lifestyle factors being shown to play a role^2^. Chromosomal aberrations, such as aneuploidies, account for up to 40–50% of all miscarriages^3^. To effectively reduce or prevent spontaneous miscarriage, studies have emphasized the importance of assessing risk factors which are modifiable—namely lifestyle and behavioural factors^4–6^. These include pre-pregnancy underweight, overweight or obesity, cigarette smoking, alcohol intake, caffeine intake and lack of dietary supplementation, such as folic acid and multivitamins, which have been found to increase the risk of miscarriage^2,4,7^. Shift work, particularly night shifts, and occupations requiring heavy lifting, have also been shown to increase the risk of miscarriage^8,9^.

Most studies that assessed pre- or early-pregnancy risk factors contributing to miscarriage were conducted during early pregnancy^1,10,11^. Recall bias could be a confounding factor especially in determining pre-pregnancy information. Given that most miscarriages occur in the early weeks of gestation, sometimes even before women recognize that they are pregnant^1^, evaluation of these factors in early pregnancy might be too late for any intervention or treatment to have an impact on the miscarriage outcome. This highlights the need to identify at-risk women prior to pregnancy for appropriate risk-modifying interventions. To date, relevant studies conducted during the preconception period have been limited^12–16^. These studies mainly focused on identifying individual risk factors for miscarriage, but not on defining the overall risk level based on the presence of multiple risk factors. Understanding the risk level (e.g. low-, intermediate-, high-risk) facilitates the design of cost-effective targeted interventions with varied intensity for those in different risk groups^17^.

Using data from the Singapore PREconception Study of long-Term maternal and child Outcomes (S-PRESTO) prospective cohort study, we aimed to develop a risk score based on a set of preconception maternal risk factors, which could be used to identify the risk levels for miscarriage among women planning to conceive. These risk factors were easily understood, readily available and did not require invasive procedures, thus increasing its acceptability and accessibility to all women of reproductive age. Most importantly, priority was given to lifestyle and behavioural factors which were modifiable, making this an attractive initiative for implementation in the general population.

## Methods

### Study participants

Between February 2015 and October 2017, 1032 women planning to conceive within the next 12 months were recruited from the general population of Singapore. Participants included women aged 18–45 years, of Chinese, Malay or Indian ethnicity (or any combination of these three ethnic groups), and who had the intention to reside in Singapore for the next 5 years. Those who reported that they were currently pregnant, had been diagnosed with Type 1 or Type 2 diabetes, had been taking anticonvulsants, oral steroids or received assisted fertility treatment in the past 1 month were not eligible. There was no active public involvement in the development of this study.

The study was conducted according to the guidelines laid down in the Declaration of Helsinki. The Singhealth Centralised Institute Review Board approved the study protocol (reference 2014/692/D). All participants provided written informed consent. This study was registered at www.clinicaltrials.gov, NCT03531658 (22/05/2018). The project is supported by the Singapore National Research Foundation under its Translational and Clinical Research (TCR) Flagship Programme and administered by the Singapore Ministry of Health’s National Medical Research Council (NMRC), Singapore—NMRC/TCR/004-NUS/2008; NMRC/TCR/012-NUHS/2014. Additional funding is provided by the Singapore Institute for Clinical Sciences, Agency for Science Technology and Research (A*STAR). The funders did not take part in the conduction of this research or writing of the present manuscript.

### Study procedure

Recruitment visits were held at the S-PRESTO cohort centre in the KK Women’s and Children’s Hospital. Research staff interviewed participants to ascertain their socio-demographic, lifestyle and health history, before conducting anthropometric measurements. Within the next 12 months, we provided participants with urinary pregnancy test kits (Biotron Diagnostics, USA), which detects the beta subunit of human chorionic gonadotropin at a detection limit of 25 IU/L, together with instructions on their use. Participants were instructed to perform a pregnancy test at home if their menstrual periods were late for 3–4 days, or 2 weeks after unprotected intercourse. If the participants have a positive urine pregnancy test, they were scheduled for an ultrasound scan to confirm pregnancy viability. In the absence of any update within 6, 9 and 12 months from the enrolment, research staff contacted participants to track their pregnancy status. Those who were lost to follow-up, who withdrew or who were non-pregnant within 12 months of enrolment were dropped from the study. Participants with confirmed pregnancies remained on follow-up until at least 16 weeks gestation.

### Study variables

We recorded each participant’s date of birth and used it to calculate the age on the day of recruitment. Age was categorized as < 30, 30–34 and ≥ 35 years^18–21^. We assessed ethnicity based on self-reported parental ethnic groups. Participants with parents of different ethnic groups were classified as mixed ethnicity. We defined history of pregnancy loss as any self-reported case of previous miscarriage, termination of pregnancy, ectopic pregnancy or stillbirth. Participants reported history of live birth > 24 weeks gestation and were classified as parous, whereas those without were classified as nulliparous. Participants were asked if they were currently smoking and were classified as yes or no. Participants were asked if they consumed any alcoholic beverage in the past three months. Based on the reported frequency and amount of consumption at each time, we calculated the total volume of alcohol intake per week, categorizing it into ≤ 250 ml and > 250 ml accordingly, for the ease of portion size estimation by the general women. We recorded data on dietary supplement intake as yes or no, including vitamins, fish oil, minerals and any type of micronutrients in the last 3 months. Probiotics and traditional medicines were not included. We measured each participant’s weight to the nearest 0.1 kg using a SECA 803 weighing machine (Hamburg, Germany) and height to the nearest 0.1 cm using a SECA 213 Portable Stadiometer (Hamburg, Germany). Body mass index (BMI) was calculated as weight (kg) divided by the square of the height (m). We classified BMI status into underweight < 18.5 kg/m^2^, normal 18.5–22.9 kg/m^2^ and overweight or obese ≥ 23 kg/m^2^ based on the World Health Organization recommended cut-off points for Asian population^22^.

### Outcome

The primary outcome was spontaneous miscarriage before 16 weeks gestation, as compared to ongoing pregnancies beyond 16 weeks gestation. We defined spontaneous miscarriage as pregnancy loss due to missed, incomplete or complete miscarriage < 16 weeks gestation^23^, when most miscarriages would have been expected to occur^24^. 16 weeks was chosen as a cut-off as miscarriages occurring before this time-point have been shown to share similar pathophysiological aetiologies, such as luteal phase deficiency, chromosomal or structural anomalies, which are different from those after 16 weeks^25,26^. In this study, diagnosis of miscarriage was based either clinically, by bleeding and obvious expulsion of an embryo or fetus, or via ultrasound^25^.

### Statistical analysis

We compared differences in participants’ baseline characteristics by pregnancy outcome of miscarriage < 16 weeks gestation and ongoing pregnancy ≥ 16 weeks gestation using the Pearson’s chi‐squared test. To construct a risk prediction model for miscarriage that could be conveniently understood and used by preconception women themselves, we considered only variables that were straightforward, easily known without prior laboratory tests, commonly reported in the literature^2,12^ and available in our dataset. These included age, ethnicity, history of pregnancy loss, parity, BMI, smoking status, alcohol intake and dietary supplement intake. For ethnicity, Chinese, Malay and mixed ethnic groups were classified together (non-Indian) and served as the reference because these ethnicities were found to have a lower incidence of miscarriage as compared with Indian.

The final predictive model was determined based on the lowest Akaike information criterion (AIC) value, which indicates a model with a better goodness of fit. We used a logistic regression model to estimate the predicted probabilities by risk score. All independent variables were in categorical forms. We assigned a score value for each variable according to the range of β coefficients, and with reference to the adjusted OR in the multivariable model^27,28^ as follows: β < 0.60 = score 1; β 0.60–0.99 = score 2; β 1.00–1.39 = score 3; β ≥ 1.40 = score 4. The lowest or absent category of each variable was set as the reference and given a score of 0, except for BMI where the normal reference range (18.5–22.9 kg/m^2^) was scored as 0, and for supplement intake where the presence of supplement intake was scored as 0. The total risk score was calculated as the sum of these individual components, with possible scores ranging from 0 to 17.

The risk score equation:

$$\begin{aligned} Miscarriage \, \,Risk\, \, Score \, & = \, 2 \, x \, \left( {Age \, \,30 - 34} \right) \, + \, 4 \, x \, \left( {Age \, \ge 35} \right) \, + \, 2 \, x \, \left( {Indian} \right) \\ & \quad + \, 2 \, x \, \left( {History\, \, of\, \, pregnancy\, \, loss} \right) \, + \, 2 \, x \, \left( {Underweight \, < 18.5 \, \,{\text{kg}}/{\text{m}}^{2} } \right) \, \\ & \quad + \, 1 \, x \, \left( {Overweight/obese \, \ge 23 \, \,{\text{kg}}/{\text{m}}^{2} } \right) \, + \, 3 \, x \, \left( {Smoker} \right) \, \\ & \quad + \, 2 \, x \, \left( {Alcohol\, \, intake \, > 250 \, \,{\text{ml}}/{\text{week}}} \right) \, + \, 2 \, x \, \left( {No\, \, dietary\, \, supplement \, \,intake} \right). \\ \end{aligned}$$

We classified the risk score into 4 categories, i.e. scores 0–3, 4–6, 7–8 and 9–17 based on the distribution of miscarriage rates in each category. We further classified them into low-, intermediate- and high-risk levels, based on the percentage of miscarriage at each category. We evaluated the discriminatory ability of the risk score model in predicting miscarriage using the area under the receiver-operating-characteristic (ROC) curves (AUC). The AUC value of > 0.70 indicates that the model exhibits a fair discriminatory ability^29^. We performed sensitivity analysis to estimate coefficients of risk factors for miscarriage by restricting data to women conceiving naturally (n = 449). Sixteen women who conceived through in vitro fertilisation or intrauterine insemination were excluded. Data were analysed using the IBM SPSS Statistic Package, version 26 (IBM Corp., Armonk, N.Y., USA) or Stata Statistical Software, Release 16 (StataCorp, College Station, TX, USA).

### Details of ethics approval

The Singhealth Centralised Institute Review Board approved the study protocol (reference 2014/692/D). All participants provided written informed consent. This study was registered at www.clinicaltrials.gov, NCT03531658 (22/05/2018).

## Results

**Figure 1:**
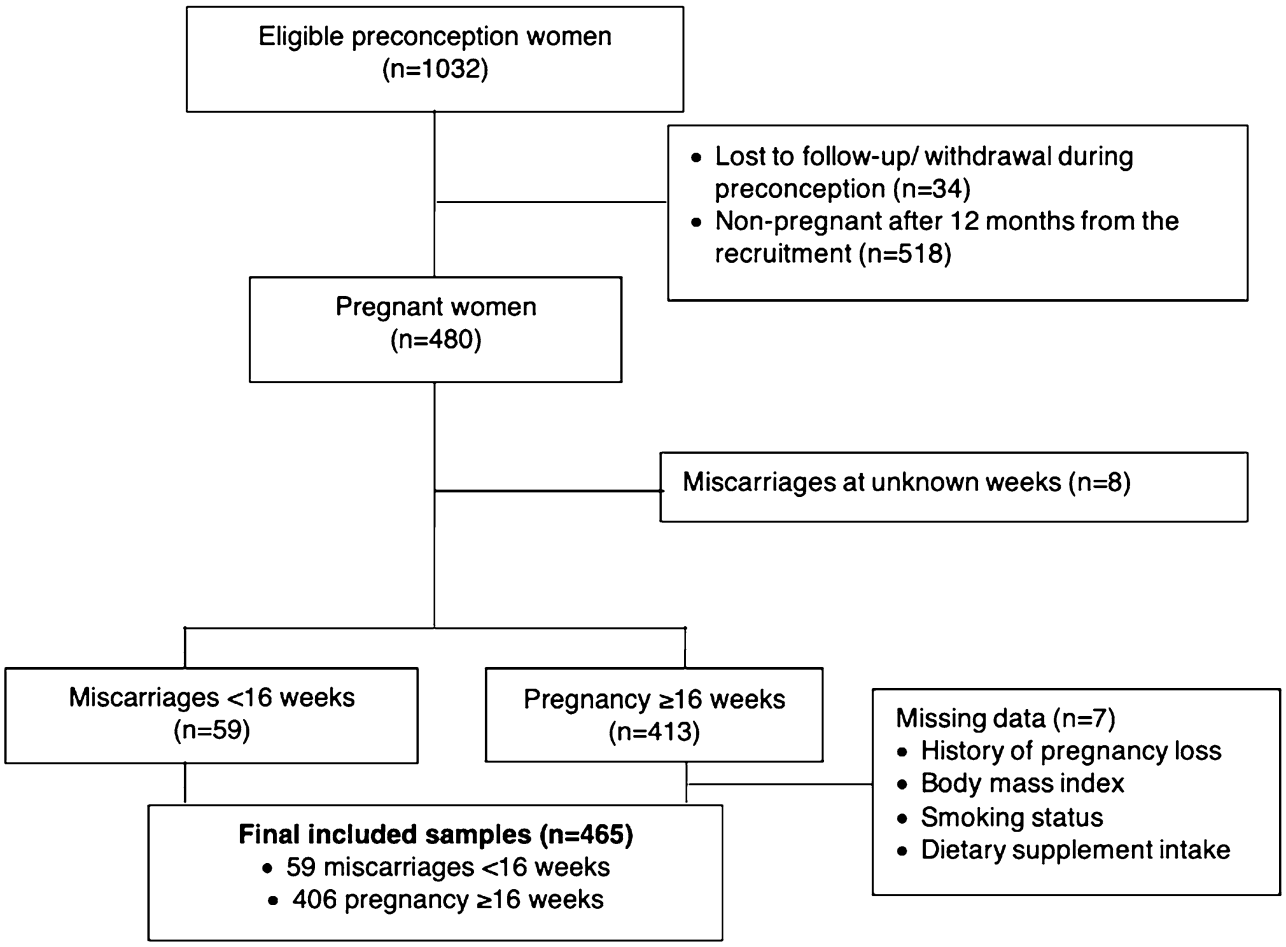
Study flowchart.

Of the women recruited preconception, 480 (46.5%) conceived within 12 months after recruitment. In this study, we only included women with a complete dataset for analysis, giving a final sample of 465 women. Among these, 59 (12.7%) had a miscarriage < 16 weeks gestation, while 406 (87.3%) had an ongoing pregnancy beyond 16 weeks gestation (Fig. 1). Compared to women who were excluded, those included were more likely to have a history of pregnancy loss (24.3% vs 20.0%), be non-smokers (96.1% vs 90.0%), have dietary supplement intake (71.0% vs 60.0%), and less likely to be overweight and obese (40.4% vs. 50.0%) (Supplementary Table S1). Compared to women who were not pregnant (n = 518), pregnant women (n = 480) were less likely to be above the age of 35 (8.3% vs 20.5%), were less likely to be overweight and obese (40.6% vs 50.9%) and more likely to have dietary supplement intake (70.7% vs 63.2%) (Supplementary Table S2).

The characteristics of women, classified based on pregnancy outcome of miscarriage and ongoing pregnancy, are shown in Table 1. Compared to women with ongoing pregnancy, those with miscarriages were more likely to be between 30 to 34 years old (55.9% vs. 49.3%) or ≥ 35 years old (16.9% vs. 6.7%), be ethnically Indian (18.6% vs. 5.9%), have a history of pregnancy loss (42.4% vs. 21.7%), be smokers (10.2% vs. 3.0%), to consume > 250 ml of alcohol per week (15.3% vs. 6.9%) and have a lack of dietary supplement intake (40.7% vs. 27.3%). Types of dietary supplementation that have been included were broadly classified as multinutrient supplement (25.5%), single nutrient supplement (47.7%) and fish oil supplement (17.3%). No significant differences in parity, BMI status, weight lifting > 25 kg and night shiftwork were observed between both groups of women.

**Table 1:** Comparison of baseline characteristics between women with miscarriages < 16 weeks gestation and with pregnancy ≥ 16 weeks, from the S-PRESTO study (n = 465).

	Total (n = 465)	Pregnancy ≥ 16 weeks (n = 406)	Miscarriage < 16 weeks (n = 59)	p^a^
**Age, n (%)**				0.004
< 30 years	195 (41.9)	179 (44.1)	16 (27.1)	
30–34 years	233 (50.1)	200 (49.3)	33 (55.9)	
≥ 35 years	37 (8.0)	27 (6.7)	10 (16.9)	
**Ethnicity, n (%)**				0.005
Chinese	351 (75.5)	310 (76.4)	41 (69.5)	
Malay	62 (13.3)	56 (13.8)	6 (10.2)	
Indian	35 (7.5)	24 (5.9)	11 (18.6)	
Mix	17 (3.7)	16 (3.9)	1 (1.7)	
**History of pregnancy loss, n (%)**				0.001
No	352 (75.7)	318 (78.3)	34 (57.6)	
Yes	113 (24.3)	88 (21.7)	25 (42.4)	
**Parity, n (%)**				0.791
Nulliparous	291 (62.6)	255 (62.8)	36 (61.0)	
Parous	174 (37.4)	151 (37.2)	23 (39.0)	
**Body mass index, n (%)**				0.057
Underweight < 18.5 kg/m^2^	30 (6.5)	26 (6.4)	4 (6.8)	
Normal 18.5–22.9 kg/m^2^	247 (53.1)	224 (55.2)	23 (39.0)	
Overweight/obese ≥ 23 kg/m^2^	188 (40.4)	156 (38.4)	32 (54.2)	
**Smoking status by number of cigarettes, n (%)**				0.004
0	447 (96.1)	394 (97.0)	53 (89.8)	
1–5	15 (3.2)	11 (2.7)	4 (6.8)	
6–10	3 (0.6)	1 (0.2)	2 (3.4)	
**Alcohol intake, n (%)**				0.027
≤ 250 ml per week	428 (92.0)	378 (93.1)	50 (84.7)	
> 250 ml per week	37 (8.0)	28 (6.9)	9 (15.3)	
**Dietary supplement intake, n (%)**				0.035
Yes	330 (71.0)	295 (72.7)	35 (59.3)	
No	135 (29.0)	111 (27.3)	24 (40.7)	
**Weight lifting > 25 kg**				0.447
No	430 (92.5)	374 (92.1)	56 (94.9)	
Yes	35 (7.5)	32 (7.9)	3 (5.1)	
**Night shiftwork**				0.581
No	416 (89.5)	362 (89.2)	54 (91.5)	
Yes	49 (10.5)	44 (10.8)	5 (8.5)	

*S-PRESTO* Singapore PREconception Study of long-Term maternal and child Outcomes.

^a^Based on Pearson’s chi-squared test.

Table 2 shows the preconception maternal risk factors associated with miscarriage. In the multivariable model, maternal age 30–34 years (odds ratio 2.22; 95% confidence interval 1.12, 4.40) or ≥ 35 years (4.59; 1.74, 12.15), history of pregnancy loss (2.37; 1.30, 4.34), cigarette smoking (3.10; 1.01, 9.54) and lack of dietary supplement intake (1.90; 1.01, 3.57) were associated with an increased odds of miscarriage. Indian ethnicity (2.30; 0.98, 5.40), underweight (2.16; 0.66, 7.11), overweight and obesity (1.73; 0.93, 3.19), alcohol intake > 250 ml per week (2.29; 0.96, 5.47) were also found to increase the odds of miscarriage, despite the associations did not reach statistical significance.

**Table 2:** Preconception risk factors for miscarriage < 16 weeks gestation, from the S-PRESTO study (n = 465).

	Model 1		Model 2		Score^a^
	Crude OR (95% CI)	Coefficient	Adjusted OR (95% CI)	Coefficient	
**Age, years**					
< 30	1.00		1.00		
30–34	1.85 (0.98, 3.47)	0.613	2.22 (1.12, 4.40)	0.797	2
≥ 35	4.14 (1.71, 10.07)	1.422	4.59 (1.74, 12.15)	1.524	4
**Ethnicity**					
Non-Indian	1.00		1.00		0
Indian	3.65 (1.68, 7.91)	1.294	2.30 (0.98, 5.40)	0.831	2
**History of pregnancy loss**					
No	1.00		1.00		0
Yes	2.66 (1.51, 4.69)	0.977	2.37 (1.30, 4.34)	0.863	2
**Body mass index**					
Underweight < 18.5 kg/m^2^	1.50 (0.48, 4.67)	0.404	2.16 (0.66, 7.11)	0.769	2
Normal 18.5–22.9 kg/m^2^	1.00		1.00		0
Overweight/obese ≥ 23 kg/m^2^	2.00 (1.13, 3.55)	0.692	1.73 (0.93, 3.19)	0.546	1
**Smoking status**					
No	1.00		1.00		0
Yes	3.72 (1.34, 10.32)	1.313	3.10 (1.01, 9.54)	1.132	3
**Alcohol intake**					
≤ 250 ml per week	1.00		1.00		0
> 250 ml per week	2.43 (1.08, 5.45)	0.888	2.29 (0.96, 5.47)	0.826	2
**Dietary supplement intake**					
Yes	1.00		1.00		0
No	1.82 (1.04, 3.20)	0.600	1.90 (1.01, 3.57)	0.640	2
Area under the ROC curve					0.74

*S-PRESTO* Singapore PREconception Study of long-Term maternal and child Outcomes, *OR* odds ratio, *CI* confidence interval, *ROC* receiver-operating-characteristic.

^a^The risk score values were estimated based on the range of β coefficients in Model 2. Score 1: < 0.60; Score 2: 0.60–0.99; Score 3: 1.00–1.39; Score 4: ≥ 1.40; Score 0: the reference category of each variable. Total score: 0–17.

**Figure 2:**
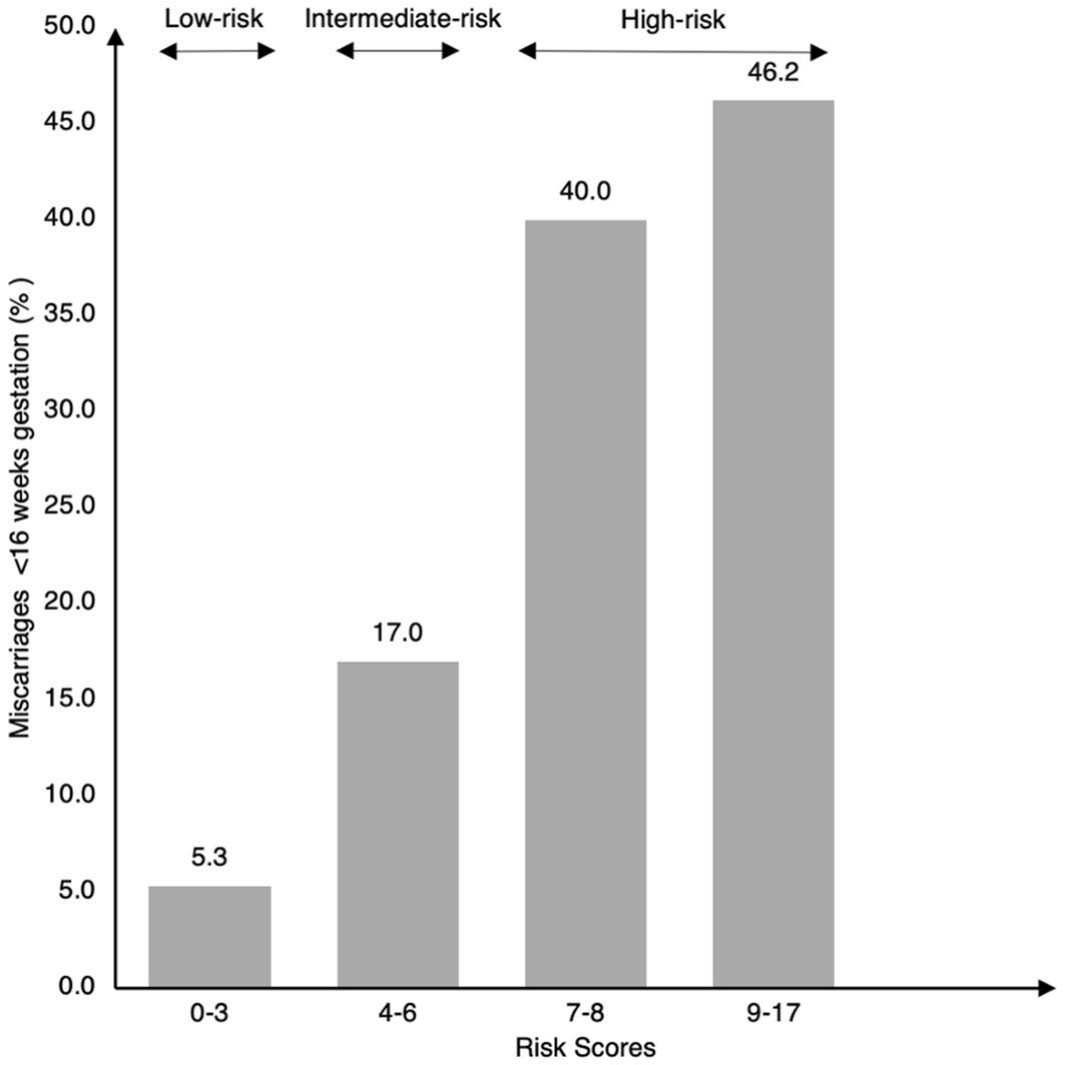
Proportion of women with miscarriages < 16 weeks gestation according to the risk scores (P-for-trend < 0.001). Scores 0–3 (< 10% miscarriage): low-risk; scores 4–6 (10 to < 40% miscarriage): intermediate-risk; scores 7–17 (≥ 40% miscarriage): high-risk.

**Figure 3:**
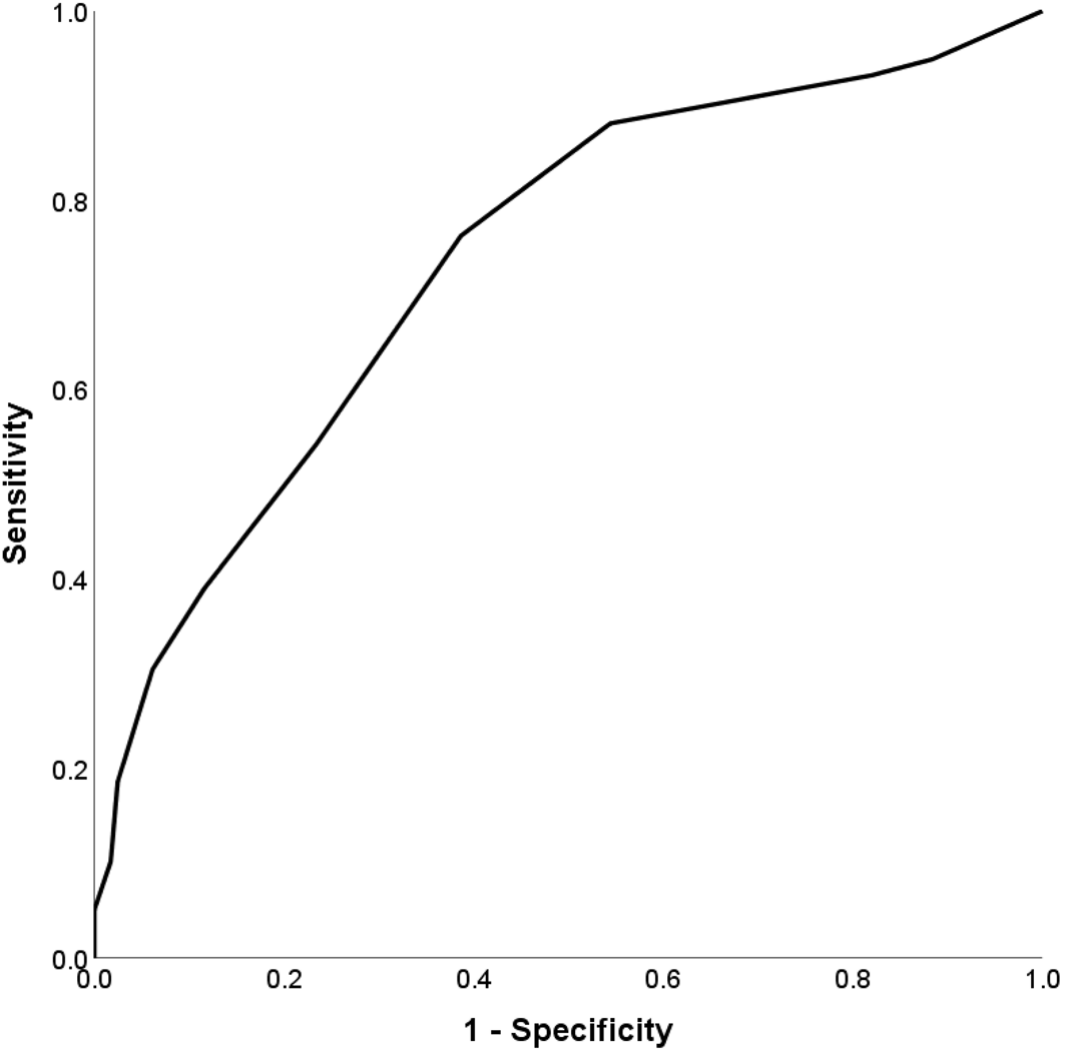
ROC curve showing the performance of the preconception miscarriage risk score in predicting miscarriage. The area under the ROC curve was 0.74 (95% confidence interval 0.67, 0.81; p < 0.001). *ROC* receiver-operating-characteristic.

Risk scores assigned for each factor in the multivariable model are presented in Table 2. The sum of risk scores ranged from 0 to 17. Out of our study population of 465, 263 (56.6%) women had a risk score of 0–3, among whom, 14 (5.3%) of them had a miscarriage; 159 (34.2%) women scored between 4 and 6, of whom 27 (17.0%) had a miscarriage. Of the 30 (6.5%) women who scored between 7 and 8, 12 (40.0%) had a miscarriage, while among the 13 (2.8%) who scored between 9 and 17, 6 (46.2%) had a miscarriage (Fig. 2). These women were then classified into three different groups, namely low-, intermediate- and high-risk levels of miscarriage. The ROC analysis revealed a fair discriminatory ability of the risk score in predicting miscarriage, as indicated by an AUC of 0.74 (95% confidence interval 0.67, 0.81; p < 0.001) (Fig. 3). When sensitivity analysis was performed by only including data for women who conceived naturally, the generated risk scores remained similar (Supplementary Table S3).

## Discussion

### Main findings

We developed a simple risk score based on a group of preconception maternal risk factors, to identify the risk levels for spontaneous miscarriage among women planning to conceive. The risk factors included were maternal age, ethnicity, history of pregnancy loss, BMI status, cigarette smoking, alcohol and dietary supplement intake during preconception. Based on the risk scores, we defined women who scored between 0 and 3 as low-risk level (< 10% of miscarriage), those who scored between 4 and 6 as intermediate-risk level (10 to < 40% of miscarriage), and those who scored between 7 and 17 as high-risk level (≥ 40% of miscarriage). Overall, the risk model showed a fair discriminatory ability to predict miscarriage < 16 weeks gestation, with an AUC of 0.74.

### Strengths and limitations

The main strength of this study is the prospective cohort design during preconception, which reduced the potential of recall bias. To our knowledge, this represents the first data-driven self-assessment preconception risk scoring tool for predicting miscarriage. However, we recognize several limitations of this study. Firstly, the risk score model was not validated using an independent dataset. This may indicate over-optimistic findings of good predictability. Secondly, the generalizability of our findings to a wider population has yet to be determined, as the current study of small sample size was confined to planned pregnancies among Asian women in Singapore. Due to the small sample size, we were also unable to perform subgroup analyses for miscarriages ≥ 16 weeks. Further, this represents a cohort of apparently healthy volunteers with 90% of participants aged < 35 years, and this may have underestimated miscarriage incidence and attenuate the estimates^30^. In addition, the ability to detect very early pregnancy losses prior to a positive urine pregnancy test may underestimate the true incidence of miscarriage. However, our study presents a proof-of-concept for preconception risk prediction models, providing a great opportunity for future validation studies to determine its utility in different populations, with further model refinement as appropriate. To increase the prediction validity, pooled data from populations with different miscarriage risk levels are required to derive common risk factors^31^. Thirdly, though dietary caffeine intake has been reported as another modifiable predictor for miscarriage^13^, we did not include it into the scoring due to the lack of data availability and the challenge in accurately quantifying caffeine intake^32^.

### Interpretation

Our cohort showed that 46.5% women spontaneously conceived after 12 cycles of pregnancy attempts, which is lower than the reported rates of greater than 70% in other studies^33–35^. However, similarly low conception rates of 42% after 12 cycles of natural conception have been previously shown in a study of Chinese women^36^. The low conception rate may help to explain the relatively low total fertility rate in Singapore of 1.10^37^. In this study, although the recruited women expressed their intention to conceive and are encouraged to engage in sexual intercourse for 2–3 times per week, however, based on the low conception rate as observed, their frequency of sexual activity may be overestimated. Owing to other issues such as a stressful lifestyle or medical conditions, it is possible that some women might have temporary stopped or delayed their pregnancy attempts during the study.

The incidence of pregnancy loss in this study was lower (12.7%) than the miscarriage rate in Singapore (16–25%)^38^. This wide variation in miscarriage could be accounted for by the higher risk of miscarriage if they presented with threatened miscarriage. It might be expected that our study would underestimate the rate of pregnancy loss due to the recruitment of healthy young volunteers who tended to be more health conscious than the general population, and thus likely to have a lower risk of miscarriage.

Among related risk factors, those modifiable ones should be targeted for intervention. These include maternal age, weight status, cigarette smoking, alcohol intake and dietary supplementation. For maternal age, our results were consistent with previous studies which showed that risk of miscarriage increased after age 30, followed by a steep increase after age 35^20,21^. This is largely due to the increased incidence of chromosome anomalies in older women^39,40^. In the risk model, assigning scores by age range emphasized the importance of initiating pregnancy planning earlier, which is a crucial message especially for young couples.

Although the association of weight status with miscarriage did not reach statistical significance in our multivariable model, its inclusion underpins the importance of maintaining optimal weight starting from preconception. Indeed, in a meta-analysis, both high and low pre-pregnancy BMI were associated with increased miscarriage risks^7^, which may be accounted for by metabolic hormonal imbalance, oxidative stress and inflammatory disturbance^7,41^. Moreover, a prospective cohort indicated that weight gain ≥ 5% over the period spanning 12–18 months preconception to 4 weeks’ gestation increased the risk of miscarriage, as compared to those who maintained their weight^41^. Weight gain during pregnancy is an important risk factor for metabolic disease later in life, and lifestyle interventions, comprising healthy diet and increased physical activity, have been shown to reduce maternal weight postpartum, weight gain during pregnancy and improved prevention of control of gestational diabetes^42^. Lifestyle interventions to optimize preconception weight has also been shown to improve cardiometabolic health years after the intervention^43^.

Cigarette smoking and alcohol intake during early pregnancy have been associated with increased miscarriage risk^1,6,10^, and this is consistent with our findings where smoking and alcohol intake during preconception also contributed to miscarriage. Nicotine found in tobacco can cause vascular spasm vasculitis, leading to placental pathology^44^; whereas alcohol, via either direct cytotoxic effects of ethanol consumption or indirect effects of alcoholism^44^, may produce deleterious effects on embryo growth. Though we showed that alcohol intake > 250 ml may increase miscarriage risk, most guidelines advocate that there is no defined safe alcohol intake levels. Some studies have reported that lack of dietary supplementation increased miscarriage risk^4,45,46^, which is in line with our findings. We did not specify the type of supplement in the risk scoring as multiple vitamins and minerals, such as folate, vitamin B6 and B12, are vital to embryonic growth and development^47^.

We showed that women with a history of pregnancy loss had an increased risk of miscarriage. This is well-defined in multiple studies that showed the risk of recurrent miscarriage was much higher in women with previous miscarriages^20,48^. Various genetic, endocrine, anatomical, immunological, thrombophilic and environmental causes have been hypothesized as etiological factors^48,49^. Several studies have demonstrated a racial and ethnicity predilection in the incidence of miscarriage^50–52^. For example, black women showed a higher risk of miscarriage than white women^50,51^. In this study, higher incidence of miscarriage was observed in Indian women compared to Chinese and Malay women. The causal mechanisms are uncertain but likely to be multifactorial, with complex interactions between genetic factors, underlying medical conditions, socioeconomic status and environmental factors^50,51^. In Singapore, Indians are considered the ethnic minority, making up 9.2% of the Singapore population, with Chinese the ethnic majority at 76.8%, Malays 13.4% and others 3.3%. However, Indians have the highest average and median household income at $7664 and $5370 respectively, followed by the Chinese at $7326 and $5370 respectively^53^. Although previous pregnancy loss and ethnicity are non-modifiable factors, their inclusion in the risk scoring would avoid underestimating the risk of miscarriage within these populations^31^. More importantly, it might increase the awareness for these women to engage in lifestyle modifications and work on modifiable risk factors to reduce the overall miscarriage risk.

Combining the aforementioned seven risk factors, we established an easily accessible risk score that could identify preconception women at high-risk of spontaneous miscarriage. This self-assessment model was designed so women could easily compute and derive their risk scores independently. It featured mainly modifiable lifestyle and behavioural factors to allow for risk modification during preconception, where it is likely to have the greatest impact. Women planning to conceive will be able to calculate their risk scores, resulting in actionable insights with an increased motivation to modify their lifestyle behaviours to achieve a successful pregnancy^54^. It may also be useful for reproductive health management for miscarriage risk reduction and prevention, by facilitating cost estimation, design of targeted interventions, assessment of women’s health literacy and lifestyle behaviour. Validation of this model in a larger independent population is warranted to verify the predictive power of the developed miscarriage risk score. Also, it is important to assess the impact of calculating these scores on women’s lifestyle practices^31^. To enhance its utility and accuracy in miscarriage prediction clinically, future studies may consider incorporating additional risk factors such as ultrasonographic features, such as the presence of fetal heart, or biochemical biomarkers such as serum progesterone^23^ and CA125^55^.

## Conclusion

The preconception period represents a great window of opportunity for intervention to reduce the risk of miscarriage. Our group developed a risk score based on seven risk factors including five modifiable and two non-modifiable factors, to stratify preconception women into different risk level subgroups for spontaneous miscarriage. The selective incorporation of only variables that can be easily determined makes it a quick and user-friendly tool for self-assessment in women of reproductive age. With increased awareness and recognition, this could empower women at higher risk of miscarriage to make lifestyle changes prior to conception around the modifiable risk factors, to reduce the overall risk of miscarriage. This should also prompt national initiatives targeted at improvements in preconception health, in a bid to optimize pregnancy and long-term health outcomes for the women and her child.

## Supplementary Information

Supplementary Tables.

## Data Availability

The data that support the findings of this study are available from S-PRESTO. Restrictions apply to the availability of these data, which were used under license for this study. Data are available from the authors with the permission of S-PRESTO.
