# Supplementary material for: Risk score to stratify miscarriage risk levels in preconception women
Source: Sci Rep. 2021 Jun 8;11:12111. doi: 10.1038/s41598-021-91567-8 (PMC8187346; doi:10.1038/s41598-021-91567-8)
Supplement: Supplementary file 1 — Supplementary Tables. [file 41598_2021_91567_MOESM1_ESM.pdf]

Risk scoring for preconception women to identify risk levels for miscarriage: a prospective cohort study

Authors: Xin Hui Choo, Chee Wai Ku, Yin Bun Cheung, Keith M. Godfrey, Yap-Seng Chong, Lynette Pei-Chi Shek, Kok Hian Tan, Thiam Chye Tan, Nadarajah Sadhana, Fabian Kok Peng Yap, Marjorelee T. Colega, Mary Foong-Fong Chong, Shiao-Yng Chan, See Ling Loy, Jerry Kok Yen Chan

Table S1 Characteristics of pregnant women based on their inclusion status in the present analysis

Characteristics	Total (n=480)	Excluded (n=15)	Included (n=465)	p[a]
Age, n (%)				0.180
<30 years	202 (42.1)	7 (46.7)	195 (41.9)	
30-34 years	238 (49.6)	5 (33.3)	233 (50.1)	
≥35 years	40 (8.3)	3 (20.0)	37 (8.0)	
Ethnicity, n (%)				0.755
Chinese	362 (75.4)	11 (73.3)	351 (75.5)	
Malay	64 (13.3)	2 (13.3)	62 (13.3)	
Indian	37 (7.7)	2 (13.3)	35 (7.5)	
Mix	17 (3.5)	0 (0.0)	17 (3.7)	
History of pregnancy loss, n (%)				<0.001
No	360 (75.8)	8 (80.0)	352 (75.7)	
Yes	115 (24.2)	2 (20.0)	113 (24.3)	
Parity, n (%)				0.259
Nulliparous	299 (62.9)	8 (80.0)	291 (62.6)	
Parous	176 (37.1)	2 (20.0)	174 (37.4)	
Body mass index, n (%)				<0.001
Underweight <18.5 kg/m^2	30 (6.4)	0 (0.0)	30 (6.5)	
Normal 18.5-22.9 kg/m^2	250 (53.1)	3 (50.0)	247 (53.1)	
Overweight/ obese ≥23 kg/m^2	191 (40.6)	3 (50.0)	188 (40.4)	
Smoking status, n (%)				<0.001
No	456 (96.0)	9 (90.0)	447 (96.1)	
Yes	19 (4.0)	1 (10.0)	18 (3.9)	
Alcohol intake, n (%)				0.255
≤250 ml per week	443 (92.3)	15 (100.0)	428 (92.0)	
>250 ml per week	37 (7.7)	0 (0.0)	37 (8.0)	
Dietary supplement intake, n (%)				<0.001
Yes	336 (70.7)	6 (60.0)	330 (71.0)	
No	139 (29.3)	4 (40.0)	135 (29.0)	

Sample sizes for total women and excluded women do not always equal to n=480 and n=15, respectively, due to missing values in the excluded samples.
[a]Based on Pearson's chi-squared test.

Table S2 Characteristics of all women based on their pregnancy status in the present analysis

Characteristics	Total (n=998)	Non-pregnant (n=518)	Pregnant (n=480)	p[a]
Age, n (%)				<0.001
<30 years	365 (36.6)	163 (31.5)	202 (42.1)	
30-34 years	487 (48.8)	249 (48.1)	238 (49.6)	
≥35 years	146 (14.6)	106 (20.5)	40 (8.3)	
Ethnicity, n (%)				0.119
Chinese	720 (72.1)	358 (69.1)	362 (75.4)	
Malay	153 (15.3)	89 (17.2)	64 (13.3)	
Indian	91 (9.1)	54 (10.4)	37 (7.7)	
Mix	34 (3.4)	17 (3.3)	17 (3.5)	
History of pregnancy loss, n (%)				0.747
No	745 (75.3)	385 (74.9)	360 (75.8)	
Yes	244 (24.7)	129 (25.1)	115 (24.2)	
Parity, n (%)				0.213
Nulliparous	642 (64.9)	343 (66.7)	299 (62.9)	
Parous	347 (35.1)	171 (33.3)	176 (37.1)	
Body mass index, n (%)				<0.001
Underweight <18.5 kg/m^2	80 (8.1)	50 (9.7)	30 (6.4)	
Normal 18.5-22.9 kg/m^2	452 (45.9)	202 (39.4)	250 (53.1)	
Overweight/ obese ≥23 kg/m^2	452 (45.9)	261 (50.9)	191 (40.6)	
Smoking status, n (%)				0.429
No	945 (95.5)	489 (95.0)	456 (96.0)	
Yes	45 (4.5)	26 (5.0)	19 (4.0)	
Alcohol intake, n (%)				0.903
≤250 ml per week	920 (92.2)	477 (92.1)	443 (92.3)	
>250 ml per week	78 (7.8)	41 (7.9)	37 (7.7)	
Dietary supplement intake, n (%)				0.012
Yes	661 (66.8)	325 (63.2)	336 (70.7)	
No	328 (33.2)	189 (36.8)	139 (29.3)	

Sample sizes do not always equal to the indicated numbers due to missing values.
[a]Based on Pearson's chi-squared test.

Table S3 Preconception risk factors for miscarriage <16 weeks gestation by including women who conceived naturally from the S-PRESTO study (n=449)

	Adjusted OR (95% CI)	Coefficient	Score[a]
Age, years			
<30	1.00		0
30-34	2.28 (1.15, 4.52)	0.824	2
≥35	5.53 (2.05, 14.91)	1.711	4
Ethnicity			
Non-Indian	1.00		0
Indian	2.18 (0.92, 5.15)	0.780	2
History of pregnancy loss			
No	1.00		0
Yes	2.31 (1.26, 4.25)	0.838	2
Body mass index			
Underweight <18.5 kg/m^2	2.06 (0.63, 6.81)	0.724	2
Normal 18.5-22.9 kg/m^2	1.00		0
Overweight/ obese ≥23 kg/m^2	1.75 (0.94, 3.24)	0.558	1
Smoking status			
No	1.00		0
Yes	3.00 (0.97, 9.25)	1.099	3
Alcohol intake			
≤250 ml per week	1.00		0
>250 ml per week	2.49 (1.03, 6.04)	0.912	2
Dietary supplements intake, n (%)			
Yes	1.00		0
No	1.81 (0.96, 3.42)	0.596	1
Area under the ROC curve			0.74

S-PRESTO, Singapore PREconception Study of long-Term maternal and child Outcomes; OR, odds ratio; CI, confidence interval; ROC, receiver-operating-characteristic.

[a]The risk score values were estimated based on the range of β coefficients. Score 1: <0.60; Score 2: 0.60-0.99; Score 3: 1.00-1.39; Score 4: ≥1.40; Score 0: the reference category of each variable. Total score: 0-16.
